# Recent Information on Pan-Genotypic Direct-Acting Antiviral Agents for HCV in Chronic Kidney Disease

**DOI:** 10.3390/v14112570

**Published:** 2022-11-20

**Authors:** Fabrizio Fabrizi, Federica Tripodi, Roberta Cerutti, Luca Nardelli, Carlo M. Alfieri, Maria F. Donato, Giuseppe Castellano

**Affiliations:** 1 Division of Nephrology, Dialysis, and Kidney Transplant, Foundation IRCCS Cà Granda Ospedale Maggiore Policlinico, 20122 Milano, Italy; 2 Department of Clinical Sciences and Community Health, University School of Medicine, 20122 Milano, Italy; 3 Division of Gastroenterology and Hepatology, Foundation IRCCS Cà Granda Ospedale Maggiore Policlinico, 20122 Milano, Italy

**Keywords:** dialysis, end-stage kidney disease, hepatitis C virus, sofosbuvir, sustained viral response

## Abstract

Background: Hepatitis C virus (HCV) is still common in patients with chronic kidney disease. It has been recently discovered that chronic HCV is a risk factor for increased incidence of CKD in the adult general population. According to a systematic review with a meta-analysis of clinical studies, pooling results of longitudinal studies (*n* = 2,299,134 unique patients) demonstrated an association between positive anti-HCV serologic status and increased incidence of CKD; the summary estimate for adjusted HR across the surveys was 1.54 (95% CI, 1.26; 1.87), (*p* < 0.0001). The introduction of direct-acting antiviral drugs (DAAs) has caused a paradigm shift in the management of HCV infection; recent guidelines recommend pan-genotypic drugs (i.e., drugs effective on all HCV genotypes) as the first-choice therapy for HCV, and these promise to be effective and safe even in the context of chronic kidney disease. Aim: The purpose of this narrative review is to show the most important data on pan-genotypic DAAs in advanced CKD (CKD stage 4/5). Methods: We recruited studies by electronic databases and grey literature. Numerous key-words (‘Hepatitis C’ AND ‘Chronic kidney disease’ AND ‘Pan-genotypic agents’, among others) were adopted. Results: The most important pan-genotypic combinations for HCV in advanced CKD are glecaprevir/pibrentasvir (GLE/PIB) and sofosbuvir/velpatasvir (SOF/VEL). Two clinical trials (EXPEDITION-4 and EXPEDITION-5) and some ‘real-world’ studies (*n* = 6) reported that GLE/PIB combinations in CKD stage 4/5 gave SVR12 rates ranging between 86 and 99%. We retrieved clinical trials (*n* = 1) and ‘real life’ studies (*n* = 6) showing the performance of SOF/VEL; according to our pooled analysis, the summary estimate of SVR rate was 100% in studies adopting SOF/VEL antiviral combinations. The drop-out rate (due to AEs) in patients on SOF/VEL ranged between 0 and 4.8%. Conclusions: Pan-genotypic combinations, such as GLE/PIB and SOF/VEL, appear effective and safe for HCV in advanced CKD, even if a limited number of studies with small sample sizes currently exist on this issue. Studies are under way to assess whether successful antiviral therapy with DAAs will translate into better survival in patients with advanced CKD.

## 1. Introduction 

HCV was identified in 1989; since then, HCV has emerged as a major public health problem [1]. According to Polaris models, there was a global prevalence of HCV RNA-positive patients of 0.7% (95% UI, 0.7%; 0.9%), corresponding to 56.8 million (95% UI, 55.2; 67.8) infections on January 1, 2020 [2]. In 2020, an estimated 641,1000 (95% CI, 623,000; 765,000) individuals started antiviral treatment. Chronic HCV infection can lead to liver cirrhosis, hepatic failure, and hepatocellular carcinoma in patients with or without kidney disease. After identification of HCV, a high frequency of serum anti-HCV antibodies and HCV ribonucleic acids (RNA) has been found in patients with chronic kidney disease [1]. 

The relationship between chronic kidney disease and HCV infection is bi-directional; HCV infection is both a cause and consequence of chronic kidney disease [1]. According to a systematic review with a meta-analysis of clinical studies, pooling results of longitudinal studies (*n* = 15 studies, *n* = 2,299,134 unique patients) demonstrated an association between positive anti-HCV serologic status and increased incidence of CKD; the summary estimate for adjusted HR across the surveys was 1.54 (95% CI, 1.26; 1.87) (*p* < 0.0001). However, between-study heterogeneity was observed (*Q* value by chi-squared test 500.3, *p* < 0.0001) [3]. 

The introduction of direct-acting antiviral agents (DAAs) has created a paradigm shift in the management of infection by HCV [1,4]. More recently, WHO recommended the adoption of pan-genotypic DAAs in order to support the campaign to eliminate HCV infection all over the world [4]. Genotype-specific DAAs have a limited antiviral spectrum of activity and frequently need ribavirin, which is commonly associated with important side-effects. Pan-genotypic regimens offer oral administration, eliminate the need for genotype testing, and provide favourable efficacy and tolerability. There is poor evidence in the medical literature concerning tolerability and effectiveness of pan-genotypic DAAs in end-stage renal disease [1]. The aim of this narrative review is to provide information on the efficacy and safety of pan-genotypic regimens of DAAs for patients with advanced CKD. 

## 2. Materials and Methods

### 2.1. Information Sources and Search Strategy

Studies were identified by searching electronic databases and sources of grey literature. The literature search was applied to PubMed MEDLINE, EMBASE, and Google Scholar. The following key words were adopted: (‘Hepatitis C Virus’ OR ‘HCV’ OR ‘Hepatitis C’) AND (‘Chronic Kidney Disease’ OR ‘End-stage kidney disease’ OR ‘Renal Insufficiency’ OR ‘Renal failure’ or ‘Renal impairment’) AND (‘Direct-acting antiviral agents’ OR ‘DAAs’ OR ‘Pan-genotypic agents’ OR ‘Sofosbuvir’ OR ‘Velpatasvir’). 

### 2.2. Statistical Methods

We performed pooled quantitative summary estimates of the sustained viral response (SVR) and discontinuation rates of antiviral therapies (pan-genotypic DAAs for HCV in advanced CKD) across individual studies using the inverse-variance method. A random-effects meta-analysis was conducted [5,6]. Outcomes were analysed on an intention-to-treat basis; all patients enrolled in these studies were included for the calculation of the response rate, whereas patients without an end-point were categorized as failures. 

Observational studies that compared the all-cause mortality in anti-HCV positive patients who received antiviral therapy compared with those who did not were also retrieved. The aRR was obtained in each study by multivariate analysis to find the impact of antiviral therapy *per se* on death rate, irrespective of the role of covariates. Pooled RRs and their 95% CIs were estimated by the weighted inverse of their variance. Heterogeneity was evaluated using Der Simonian and Laird’s *Q* test and quantified by calculating the proportion of the total variance attributable to between-study variance (R*i*). We computed both fixed- and random-effects models but used the latter in case of large heterogeneity. We adopted HEpiMA, a novel software program that carries out a complete study of heterogeneity of study effects [6]. Novel and useful estimators of heterogeneity, such as R*_i_* and CV_B_ were used. Heterogeneity was considered substantial if R_*i*_ was ≥ 0.75. A two-sided *p*-value of <0.05 was considered statistically significant. 

## 3. Results

### 3.1. Epidemiology

Abundant information exists on the epidemiology of hepatitis C virus infection in patients with end-stage renal disease. In 2012–2015, anti-HCV antibody prevalence among prevalent HD patients in the DOPPS was 9.9% overall (21 countries all over the world); it ranged from 4.1% in Belgium to 20.1% across the Gulf Cooperation Council Countries (GCCC; Bahrain, Kuwait, Oman, Qatar, Saudi Arabia, and United Arab Emirates), whereas >8% of patients receiving HD in China, Italy, Japan, Russia, and Spain were anti-HCV positive [7]. 

Another survey was conducted among Medicare beneficiaries with HCV undergoing haemodialysis in the United States (2005–2016). A total of 291,663 patients on haemodialysis were enrolled. The prevalence of HCV in patients on haemodialysis was greater than in individuals not on HD (4.2 vs. <1%) [8]. 

Evidence on the prevalence and incidence rates of HCV among patients on long-term dialysis in the emerging world was not satisfactory—several studies with small sample sizes have been published, and recorded prevalence rates of up to around 80% [9,10,11,12]. Some systematic reviews have been made on this point; Harfouche et al. collected data from 289 studies (*n* = 106, 463 unique patients) and found a regional pooled mean estimate of 29.2% (95% CI, 25.6%; 32.8%) for HCV antibody prevalence among patients on long-term haemodialysis in the Middle East and North Africa (MENA) [10].

### 3.2. Natural History of HCV Infection 

It is difficult to make a detailed evaluation of the natural history of HCV infection in patients with chronic kidney disease, particularly those on maintenance dialysis. Various reasons explain this—the natural history of HCV usually spans decades in patients with intact kidneys, whereas dialysis patients have limited life expectancies. In fact, patients with chronic kidney disease have higher morbidity and mortality than the general population, due to aging and comorbidities. HCV infection is frequently asymptomatic, with an apparently indolent course even in patients with advanced chronic kidney disease. Aminotransferase values are lower in patients on maintenance dialysis; thus, it is not easy to recognise the occurrence of liver disease on the grounds of biochemical abnormalities. A total of 506 patients undergoing regular dialysis in northern Italy (Lombardy) were tested by anti-HCV ELISA and PCR assays for the detection of anti-HCV antibodies and HCV RNA in serum, respectively. Serum transaminase values were significantly greater in HCV RNA-positive than HCV RNA-negative patients, 19.3 ± 1.6 vs. 15.7 ± 1.6 (*p* = 0.008) and 22.8 ± 1.7 vs. 16.1 ± 1.7 (*p* = 0.0001). According to logistic regression analysis, detectable HCV RNA in serum was a strong predictor of raised AST (*p* = 0.0001) and ALT (*p* = 0.000001) values [13]. 

The current availability of direct-acting antiviral agents, which are considered to be safe and have high efficacy, precludes the implementation of large observational studies and prolonged follow-up to assess the course of chronic HCV infection in end-stage kidney disease. It has been stated that survival in most patients with stage 1 and 2 CKD is not different from that observed in the general population with intact kidneys. Survival in patients with CKD stages 3–5 is lower than that observed in the general population, and some information has been recently accumulated on the link between positive anti-HCV serologic status and the death rate in the dialysis population. Death can be considered a reliable endpoint in the context of observational studies evaluating the course of HCV over time in patients with intact kidneys or end-stage kidney disease, and some clinical studies have carried out such analyses. We recently conducted a systematic review with a meta-analysis of observational studies (*n* = 23 studies; *n* = 574,081 patients on long-term dialysis). We found that positive anti-HCV serologic status was an independent and significant risk factor for death in the dialysis population. The overall estimate for adjusted mortality (all-cause death risk) with HCV was 1.26 (95% CI, 1.18; 1.34) (*p* < 0.0001) [14]. We performed stratified analyses to assess the causes of the increased death risk. The summary estimate for adjusted mortality (liver disease-related mortality) was 5.05 (95% CI, 2.53; 10.0) (*p* < 0.0001). The overall estimate for cardiovascular death risk was 1.18 (95% CI, 1.085; 1.29) (*p* < 0.0001). Using meta-regression, we observed that the relationship between positive anti-HCV serologic status and all-cause death risk was more evident in surveys with a larger size (*p* < 0.0001), higher proportion of diabetics (*p* = 0.0005), and HCV-positive patients (*p* = 0.001) [14]. 

The latest report on this issue was published a few months ago by Ko and coworkers. After adjusting for potential confounding factors, the multivariate Cox regression resulted in a significant association between serum HCV RNA-positive status and death rate in a cohort of haemodialysis patients who started chronic HD after 2002 (AHR, 1.48; 95% CI, 1.13; 1.93, *p* = 0.005). The study group in such a report was large (*n* = 1,437 patients on maintenance HD in Hiroshima, Japan) [15]. 

### 3.3. Antiviral Therapy of HCV and Its Purpose (Pan-Genotypic Regimens)

The purpose of antiviral therapy of HCV was the achievement of SVR12. SVR12 is the elimination of HCV RNA from serum which persists at least 12 weeks after completing antiviral therapy [16]. Antiviral treatment was indicated for patients showing anti-HCV antibody and detectable HCV RNA in serum. Solid evidence in the medical literature exists, suggesting that the achievement of SVR12 (‘the cure’) was associated with better survival and quality of life in patients with intact kidneys [17]. Patients with chronic kidney disease, HCV/HIV co-infection, and HCV/HBV co-infection who have had previous unsuccessful DAA regimens were defined as “special populations”, where the antiviral approach was biased by low efficacy and safety (due to high rate of co-infections and comorbidities). It appears now that DAAs have abolished the notion of ‘special populations’ and pan-genotypic regimens promise to be effective and safe, even in this context. Pan-genotypic agents need only minimal monitoring and this encourages a test-and-treat approach as the focus of HCV management moves toward global elimination (with simplified protocols).

### 3.4. Natural History of HCV, HD Population, and Antivirals

The evidence in the medical literature supporting the antiviral treatment of HCV in patients receiving long-term dialysis is poor. Prior to the advent of DAAs, IFN-based regimens were the standard of care for the antiviral treatment of HCV. Clinicians had been reluctant to adopt IFN-based regimens for HCV in patients on maintenance dialysis, due to limited efficacy and low tolerability of IFN-based regimens in the haemodialysis setting. The effectiveness and tolerability of combined antiviral therapy (pegylated interferon plus ribavirin) in patients on regular dialysis had been addressed in a meta-analysis of clinical studies. We identified eleven clinical studies (*n* = 287 unique patients) and the summary estimate for SVR and drop-out rate was 0.60 (95% CI, 0.47; 0.71) and 0.18 (95% CI, 0.08; 0.35), respectively. The most common source of drop-out was anaemia (11/46 = 23%) [18]. The limited efficacy and tolerability of combined antiviral therapy in the dialysis population meant that only a minority of dialysis patients were ‘cured’ with such approach. 

To date, a few studies with small sample sizes have recorded higher survival rates in haemodialysis patients who received antiviral therapy compared with HCV-infected patients on HD who did not receive it. Goodkin and colleagues [19] evaluated the Dialysis Outcomes and Practice Patterns Study; 49,762 patients on long-term haemodialysis in 12 countries were enrolled (1996–2011), and survival and other clinical parameters were reviewed over a median 1.4 year per study phase. There were 4,735 (9.5%) patients with HCV infection and 4589 (96.9%) of them had a history of a prescription of antivirals. In the group of HCV-infected patients with an overlapping propensity for antiviral treatment, there was no difference regarding the death rate among patients who received antiviral treatment who died and those who did not (Table 1). The adjusted mortality risk (aHR for mortality) was 0.47 (95% CI, 0.17; 1.26, NS).

Soderholm and coworkers [20] gave antiviral treatment to 45 of 268 patients on long-term haemodialysis with chronic HCV. Antiviral treatment was associated with a favourable outcome; the death rate was lower during the study period for treated patients than for untreated patients (*p* = 0.0001) (Table 1). According to their multivariate analysis, kidney transplant (aOR, 2.97, 95% CI, 1.64; 5.37, *p* = 0.0001), acute kidney failure before renal replacement therapy (aOR, 2.518, 95% CI, 1.39; 4.54, *p* = 0.002), and antiviral treatment (aOR, 3.54, 95% CI, 1.63; 7.79, *p* = 0.001) were independent predictors of improved survival in patients on maintenance haemodialysis with chronic HCV. Age at HD initiation was linked to lower survival (aOR, 0.968, 95% CI, 0.94; 0.98, *p* = 0.004). 

Hsu and colleagues [21] worked on the National Health Insurance program in Taiwan and investigated whether interferon-based treatment was associated with improved survival in ESRD with HCV infection. In their cohort of HCV-infected patients (*n* = 2231), 134 (6.01%) patients received interferon and 2097 (93.9%) did not; the mean follow-up duration was 3.22 years (Table 1). The aHR for mortality was 3.91 (95% CI, 0.54; 28.1) in the untreated HCV cohort. In the subset of patients with HCV and without cirrhosis, patients who did not receive IFN-based therapy had a greater risk of death in comparison with the treated group (aHR, 6.31, 95% CI, 1.57; 25.4). In the subgroup of HCV-infected patients with cirrhosis and/or liver cancer, no differences in the risk of death occurred between those who received IFN or not (NS). 

Chen and coworkers [22] made a large nationwide retrospective cohort study (*n* = 93,894 Taiwanese adults diagnosed with stage 1–5 CKD and without HBV infection) and used propensity score-matched and competing risk analyses to evaluate the long-term patient survival (death rate) of anti-HCV therapy, especially interferon-based therapy, in CKD patients. They observed that the treated cohort had a 29% (95% CI, 0.54; 0.92) (*p* = 0.011) decrease in death compared with the untreated cohort. 

The last report on this topic was published by Perez de Josè and coworkers [23]. They retrieved a large cohort of patients with HCV-related mixed cryoglobulinemia. At baseline, mean serum creatinine and GFR were 1.4 mg/dL and 56 mL/min, respectively, and mean proteinuria was 2.1 gr/day. Overall, 100 patients underwent antiviral therapy with DAAs, 24 with IFN plus ribavirin, and 15 remained untreated. The death rate was greater in those patients who did not receive antiviral therapy (Table 1) compared with patients who underwent therapy for HCV; patients treated with DAAs had reduced mortality, aHR, 0.12 (95% CI, 0.04; 0.40, *p* < 0.001).

**Figure 1:**
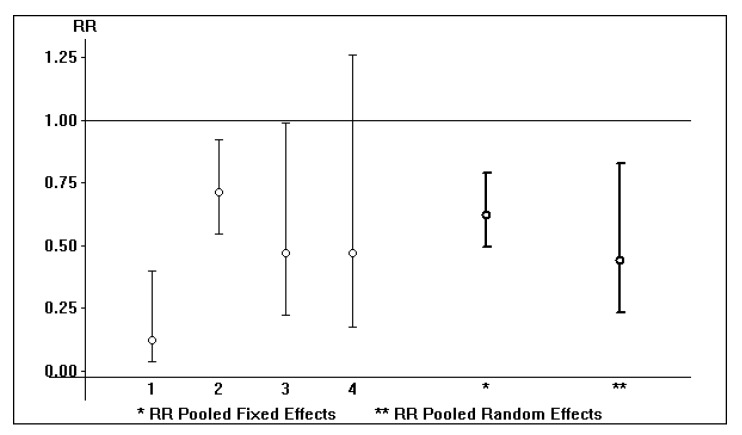
The impact of antiviral therapy on death rate in patients with advanced CKD: pooled adjusted RR according to fixed- and random-effects models.

All the studies reported in Table 1 performed multivariate analysis in order to assess a significant and independent relationship between death rate and antiviral therapy. On the grounds of our meta-analysis of observational studies (random-effects model) [5,6], the pooled adjusted RR was 0.66 (95% CI, 0.52; 0.84, *p* < 0.001) (Figure 1). The results obtained with the fixed-and the random-effects models were similar (Figure 1); however, there was some heterogeneity (R*_i_* = 0.82, CV_between 1.07). Further study is needed to make more definitive conclusions. 

### 3.5. Pan-Genotypic DAAs (Sofosbuvir) 

**Figure 2:**
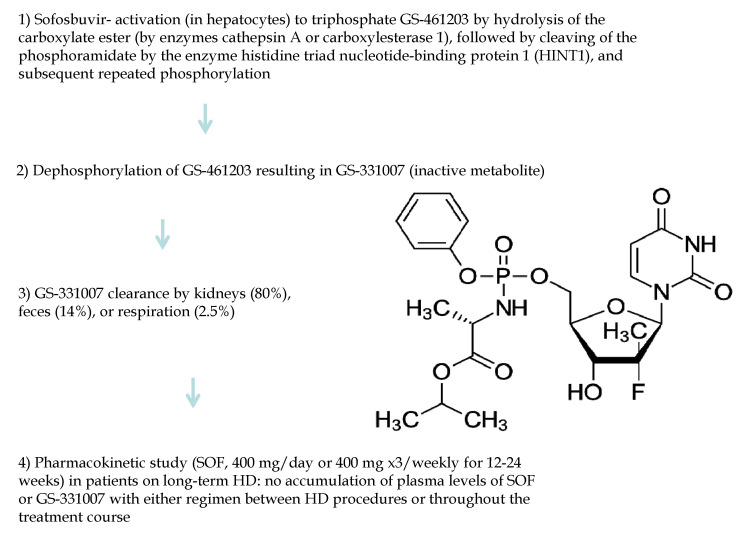
Sofosbuvir: metabolism in advanced kidney impairment and structure.

Sofosbuvir was approved in 2013 and is now the backbone of the most commonly used DAA regimens. SOF is an oral nucleoside analogue and potent inhibitor of the NS5B RNA-dependent RNA polymerase. Upon oral administration, SOF is metabolized (at liver level) to 2′-deoxy-2′-alpha-fluoro-beta-C-methyluridine-5′-monophophate, which undergoes conversion to the active triphosphate form (GS-461203). SOF acts as a HCV RNA chain terminator by inhibiting NS5B RNA-dependent RNA polymerase, which is essential for the replication of the HCV RNA viral genome (Figure 2). The dephosphorylation of GS-461203 produces an inactive metabolite (GS-331007) that undergoes large clearance by the kidneys. The administration of a single full-dose of SOF reported a greater plasma AUC in individuals with CKD stage 5 (1.33-fold) and CKD stage 4 (2.73-fold) than in patients with an estimated GFR > 80 mL/min/1.73 m^2^. In addition, the administration of a single full dose of SOF revealed that the plasma AUC of GS-331007 were 5.6-fold and 6.83-fold higher in patients with CKD stages 4 and 5, respectively, than those with intact kidneys [24]. A pharmacokinetic study with SOF at a dose of 400 mg per day or 400 mg thrice weekly for 12–24 weeks in patients undergoing long-term haemodialysis did not result in SOF accumulation between HD sessions or throughout the treatment course [25]. At the beginning, SOF use had not been recommended in patients with end-stage renal disease due to the fear of accumulation of SOF or its active metabolites. Since then, several studies reported satisfactory efficacy and tolerability regarding SOF use in advanced CKD. In November 2019, the US FDA amended the package inserts for sofosbuvir-containing regimens to allow use in patients with renal disease, including those with CKD stage 4 and 5 and those on dialysis. 

A systematic review of the medical literature with a meta-analysis of clinical studies has been recently published with the aim to assess the effectiveness and tolerability of SOF-based regimens in patients with advanced CKD (CKD stage 4 and 5, including patients receiving long-term haemodialysis). The primary end-point was the frequency of sustained viral response (as a measure of efficacy); the secondary outcomes were the rates of SAEs and drop-out due to AEs (as measures of tolerability). We identified 30 clinical studies (*n* = 1537 unique patients). The overall frequency of SVR12 was 0.99 (95% CI, 0.97; 1.0, *I*^2^ = 99.8%); the pooled frequency of SAEs was 0.09 (95% CI, 0.05; 0.13, *I*^2^ = 84.3%). Some (*n* = 6; 69 unique patients) clinical studies reported eGFR values at the beginning and end of antiviral therapy, and no consistent changes were recorded [26]. The conclusion of the authors was that SOF-based regimens appear safe and effective even in patients with CKD stage 4 and 5. Serum creatinine levels should be carefully monitored during therapy with SOF in the CKD population [26]. 

### 3.6. Pan-Genotypic DAAs (Glecaprevir/Pibrentasvir)

EXPEDITION-4 was a phase III multi-center open-label trial to assess the efficacy and safety of antiviral therapy for HCV (combined therapy of the NS3/4A protease inhibitor glecaprevir and the NS5A inhibitor pibrentasvir) for 12 weeks in adults with HCV infection and HCV genotype 1, 2, 3, 4, 5, or 6 and compensated liver disease (with or without cirrhosis) [27]. All patients in the study group had chronic kidney disease stages 4/5 (dialysis-dependent or not). The primary endpoint was the sustained viral response, 12 weeks after the end of treatment. There were 104 patients in the study group, 52% had genotype 1 infection, 16% had genotype 2 infection, 11% had genotype 3 infection, 19% had genotype 4 infection, and 2% had genotype 5 and 6 infections. The frequency of SVR was 98% (102/104) (95% CI, 95%; 100%), according to ITT analysis. Two patients failed to achieve SVR12 because of early discontinuation and loss to follow-up. SAEs were noted in 24% (25/104) of patients; none of the SAEs were considered by the study investigators to be drug related. Four patients discontinued the study due to adverse events, three of them had SVR. Some (20%, 21/104) patients complained of itching. EXPEDITION-5 is a phase III trial aimed to assess efficacy and safety of glecaprevir/pibrentasvir by oral route (300/120 mg daily, consisting of three tablets of 100/40 mg each), once a day for 8 to 16 weeks. A total of 101 patients with CKD stages 3b–5 were recruited. Fifty-five per cent of patients had HCV genotype 1 infection, 27% had genotype 2 infection, 15% had genotype 3 infection, and 4% had genotype 4; there were no patients with HCV genotype 5 or 6 infections. The SVR12 rate was 97% (98/101, 95% CI, 91.6; 99) by ITT. Serious AEs were reported in 12% of patients; none were related to the study drug [28]. 

Some ‘real-life’ studies have also been published in patients with advanced CKD and showed SVR12 rates similar to those observed in EXPEDITION-4 and EXPEDITION-5 trials (Table 2) [29,30,31,32,33,34]. The most common side-effect was pruritus (range, 0–61%), but it was mild in most patients. The frequency of AEs resulting in discontinuation of therapy was extremely low (Table 2). The most frequent causes of discontinuation of therapy due to AEs were pruritus (*n* = 3) and raised serum creatinine (*n* = 2). Other reasons were cerebral infarction (*n* = 1), fungal peritonitis (*n* = 1), patient preference (*n* = 1), and cardiomyopathy (*n* = 1). 

### 3.7. Pan-Genotypic DAAs (Sofosbuvir/Velpatasvir)

Borgia et al. performed a phase II single-arm study to treat 59 patients with genotype 1–6 HCV infection on long-term haemodialysis (*n* = 54) or peritoneal dialysis (*n* = 5) [35]. These patients received SOF/VEL (400/100 mg) once daily for 12 weeks. HCV-infected patients who were treatment-naïve or treatment-experienced with compensated cirrhosis or without cirrhosis were included. Out of 59 patients, 56 achieved SVR12 (95% CI, 86–99%). There were three patients who did not achieve SVR12: two had virological relapse (at post-treatment 4) and one of them prematurely discontinued antiviral treatment. Another patient died from suicide after obtaining SVR12. Most patients experienced an adverse event (80%), the majority of which were mild or moderate in severity. No patients prematurely discontinued SOF/VEL due to AEs. 

Some real-world studies [36,37,38,39,40,41,42] and meta-analyses [43] have been published regarding SOF-VEL for HCV in ESRD (Table 3 and Table 4). The SVR12 rates ranged between 89 and 100% in patients with advanced CKD, according to ITT analysis. Our pooled analysis of clinical studies showed that the summary estimate of SVR rates after pan-genotypic antiviral therapy (SOF/VEL) in ESRD was 1.0 (95% CI, 1.0; 1.0) (Table 5). The most common adverse event was nausea (reported in five studies), followed by headache (in three reports). Some patients (*n* = 7) showed early discontinuation of therapy due to AEs, and the causes were weakness (*n* = 1), anaemia (*n* = 1), dizziness (*n* = 1), and gastrointestinal disorders (*n* = 4). 

No data have been currently published on the antiviral combination of sofosbuvir/velpatasvir/voxilaprevir (SOF/VEL/VOX) in patients with advanced CKD. 

## 4. Conclusions

Patients with advanced CKD have been defined a ‘special population’ or ‘difficult-to-treat population’, where antiviral therapy has historically been unable to generate high efficacy and safety. DAA-based regimens have revolutionized the management of HCV, and recommended DAA regimens can vary in duration, dosing frequency, pill burden, and co-administration of ribavirin. Pan-genotypic drugs allow the simplification of the management of HCV. These drugs reduce the need for pre-treatment testing, and consequently, the time between HCV diagnosis and therapy initiation is shortened. Pan-genotypic agents promise to be safe and effective, even in patients with advanced CKD, and more studies are needed to confirm and expand such pieces of evidence. An important limitation of the present narrative review was the limited number of included studies and the small number of patients overall. Research aimed to assess whether successful antiviral therapy with DAAs will improve survival of patients with advanced CKD is in progress. 

## Figures and Tables

**Table 1:** The impact of antiviral therapy upon survival in HCV-positive patients who received antiviral therapy: univariate analysis.

Study	Reference Year	Death Rate (Treated)	Death Rate (Untreated)	*p*	Country
**Goodkin D, *et al.***	2013	4/42 (9.5%)	638/3,307 (21%)	NS	USA
**Hsu Y, *et al.***	2015	7/134 (5.2%)	581/2,097 (27.7%)	0.0001	Taiwan
**Soderholm J, *et al.***	2018	11/45 (24%)	124/223 (56%)	0.0001	Sweden
**Chen Y, *et al.***	2019	61/482 (12.7%)	648/1,928 (33.6%)	0.0001	Taiwan
**Perez de Josè A, *et al.***	2021	13/124 (10.5%)	10/15 (67%)	0.0001	Spain

**Table 2:** Pan-genotypic agents (GLE/PIB) for HCV in advanced CKD: real-life studies.

Study	Reference Year	SVR Rate	AEs Resulting in Drug Discontinuation	Country	Study Design
**Suda G, *et al.***	2019	26/27 (96.3%)	2 (7.4%)	Japan	Prospective
**Atsukawa M, *et al.***	2019	140/141 (99.3%)	3 (7.2%)	Japan	Prospective
**Yen H, *et al.***	2020	42/44 (95.5%)	1 (2.3%)	Taiwan	Retrospective
**Yap D, *et al.***	2020	18/21 (85.7%)	1 (4%)	Hong Kong/Taiwan	Prospective
**Liu C, et al.**	2020	107/108 (99%)	2 (3%)	Taiwan	Retrospective
**Stein K, *et al.***	2022	29/33 (87.9%)	0%	Germany	Prospective

**Table 3:** Studies on SOF/VEL in advanced CKD: characteristics of study patients (and viral response).

Study	Reference Year	Study Size	SVR Rate	Age, years	Males, *n*	Country
**Borgia S, *et al.***	2019	59	56/59 (94.9%)	60 (33; 91)	35 (59%)	Canada
**Gohel K, *et al.***	2020	3	3/3 (100%)	46.5	NA	India
**Mostafi M, *et al.***	2020	44	44/44 (100%)	43.7 ± 12	NA	Bangladesh
**Gaur N, *et al.***	2020	31	30/31 (96.8%)	39.8 ± 10.8	24 (77.5%)	India
**Yu M, *et al.***	2021	105	94/105 (89.5%)	66.2 ± 10	54 (51.4%)	Taiwan
**Taneja S, *et al.***	2021	51	49/51 (96%)	42.8 ± 14.6	41 (80.4%)	India
**Liu C, *et al.***	2022	191	181/191 (94.8%)	65 (23; 95)	104 (54.5%)	Taiwan

**Table 4:** Studies on SOF/VEL in advanced CKD: characteristics of study patients (and drop-out rate).

Study	HBsAg, *n*	HCV Genotype 1, *n*	Cirrhosis, *n*	Treatment-Naïve, *n*	Diabetics, *n*	Drop-Out Rate (Due to AEs), *n*
**Borgia S, *et al.***	NA	27 (45.8%)	17 (28.8%)	46 (77.9%)	19 (32%)	0
**Gohel K, *et al.***	NA	NA	0	3 (100%)	NA	0
**Mostafi M, *et al.***	0	NA	10 (23%)	44 (100%)	28 (63.6%)	0
**Gaur N, *et al.***	6 (19.3%)	21 (67.7%)	3 (9.6%)	31 (100%)	6 (19%)	0
**Yu M, *et al.***	8 (7.6%)	46 (43.8%)	37 (35.2%)	NA	65 (61.9%)	5/105 (4.8%)
**Taneja S, *et al.***	NA	15 (79%)	10 (19.6%)	43 (84.3%)	NA	0
**Liu C, *et al.***	5 (2.6%)	112 (58.6%)	27 (14.1%)	175 (91.6%)	NA	2/191 (1%)

**Table 5:** Pooled SVR rate after antiviral therapy with pan-genotypic DAAs (SOF/VEL) in advanced CKD. Test for heterogeneity: Chi^2^ = 32.13, df = 6 (*p* < 0.0001), *I*^2^ = 81.3%. Test for overall effect: Z = 2716.6 (*p* < 0.0001).

	Weight (%)	SVR Rate (SE)	SVR Rate (Random-Effects Model) 95% CI	Year
**Borgia A, *et al.***	0.02	0.94 (0.0286)	0.95 (0.89; 1.01)	2019
**Gohel K, *et al.***	49.95	1 (0.0001)	1.0 (1.00; 1.00)	2020
**Mostafi M, *et al.***	49.95	1 (0.0001)	1.0 (1.00; 1.00)	2020
**Gaur N, *et al.***	0.01	0.96 (0.0352)	0.96 (0.89; 1.03)	2020
**Yu M, *et al.***	0.01	0.89 (0.0305)	0.89 (0.83; 0.95)	2021
**Taneja S, *et al.***	0.02	0.96 (0.0270)	0.96 (0.91; 0.97)	2021
**Liu C, *et al.***	0.05	0.94 (0.0170)	0.94 (0.91; 0.97)	2022
**Total (95% CI)**	100.00		1.0 (1.00; 1.00)	

## Data Availability

Not applicable.

## Abbreviations

**AH**	Arterial hypertension
**AUC**	Area under curve
**CI**	Confidence intervals
**CKD**	Chronic kidney disease
**CLD**	Chronic liver disease
**COPD**	Chronic obstructive pulmonary disease
**CVD**	Cardiovascular disease
**DAAs**	Direct acting antiviral agents
**DM**	Diabetes mellitus
**eGFR**	Estimated glomerular filtration rate
**ESRD**	End-stage renal disease
**GLE**	Glecaprevir
**HBsAg**	Hepatitis B virus antigen
**HCV**	Hepatitis C virus
**HCW**	Health care worker
**HD**	Haemodialysis
**IFN**	Interferon
**ITT**	Intention-to-treat
**KDIGO**	Kidney Disease: Improving Global Outcomes
**NA**	Not available
**PD**	Peritoneal dialysis
**PIB**	Pibrentasvir
**RRT**	Renal replacement therapy
**SAE**	Severe adverse event
**SE**	Standard error
**SOF**	Sofosbuvir
**SVR**	Sustained virological response
**VEL**	Velpatasvir
**VOX**	Voxilaprevir
**WHO**	World Health Organization
